# Molecular, morphological and fossil input data for inferring relationship among viviparous brotulas (Bythitidae) – Resulting in a family status change for Dinematichthyidae

**DOI:** 10.1016/j.dib.2016.05.055

**Published:** 2016-05-30

**Authors:** Steen Wilhelm Knudsen, Peter Rask Møller, Werner Schwarzhans, Jørgen G. Nielsen

**Affiliations:** Natural History Museum of Denmark, University of Copenhagen, Universitetsparken 15, DK-2100 Copenhagen Ø, Denmark

**Keywords:** Bythitinae, Aphyonidae, pedomorphism, Coral reef fishes, Deepsea fishes, Cave fishes

## Abstract

This article comprise the data related to the research article (Møller et al., 2016) [1], and makes it possible to explore and reproduce the topologies that allowed [1] to infer the relationship between the families Bythitidae and Dinematichthyidae. The supplementary data holds nexus-input files for the Bayesian analysis and the ‘.xml’-input files – with and without nucleotide data – that are used in the fossil-calibrated phylogenetic analysis with a relaxed clock model. The resulting topologies are provided as ‘.new’-files together with a characters matrix file for traits to trace across the inferred phylogenies.

**Specifications Table**

**Table:** 

Subject area	Biology, Genetics and Genomics
More specific subject area	Phylogenetics and Phylogenomics
Type of data	*Phylogenetic.tre,.nex and.xml files*
How data was acquired	*The sequence reads were examined using Sequencher v. 4.0 (sequence analysis software, Gene Codes Corporation, Ann Arbor, MI USA http://www.genecodes.com) and Geneious v. R7*[2]*, as described in the materials and methods section. Alignment was performed with MAFFT*[3]*, and optimal partitioning and substitution models was inferred using PartitionFinder*[4]*. The level of nucleotide substitution saturation was inferred with DAMBE*[5]*. The resulting data matrix was analyzed in MrBayes v.3.2.*[6]*and BEAST v.1.8.0*[7]*, with log-files examined in Tracer*[8]*and resulting topologies examined in FigTree v. 1.4.2*[9]*and Mesquite v.3.04*[10].
Data format	*Analyzed*
Experimental factors	*Sequence reads were visually inspected in Sequencher and Geneious. Alignements prepared in MAFFT*[3]*were inspected for nucleotide saturation in DAMBE*[5]*. Third codon positions in the mtDNA-nd4 fragment was removed from the alignment, as there appeared to be substantial saturation on these nucleotide positions – see*[1]*for additional details. The phylogenies inferred was found using the substitution models inferred in PartionFinder, and can also be found in the ‘.nex’- and ‘.xml’-files provided. The fossil-calibrations applied can be found in both the ‘.xml’-files provided, the supplied BEAuti-file and in*Table 3*in*[1].
Experimental features	*Tissue samples was obtained from 30 species of Ophidiiformes and used for DNA extractions, PCR amplification and Sanger Sequencing. Sequence reads from mitochondrial DNA and nuclear DNA was visually inspected and assembled in alignments that subsequently could be used for preparing nexus-input files for analysis in MrBayes and BEAST to infer the evolutionary relationship among Ophidiiformes. Nucleotide sequences from extant outgroup representative species from Beryciformes, Cetomimiformes, Gadiformes, Gasterosteiformes, Lampriformes and Perciformes (listed in*Table 3[1]*) allowed nodes in the topology to be time-calibrated using similar settings as described by*[11]*. The calibrations applied are also listed in*Table 3*by*[1].
Data source location	*n/a*
Data accessibility	*Data are with this article*

**Value of the data**

● The provided nexus-files holds sequence data and alignments and can be directly utilized in future studies in the evolution of perciform fishes
● The nexus file with traits can be used to reconstruct and trace ancestral characters states across inferred phylogenies.
● The provided ‘.xml’-files and the BEAuti-file holds information on the settings applied in the fossil-calibrated analysis, which can facilitate similar fossil-calibrated studies on perciform fishes.

## 1 Data

Sequence alignments are provided in ‘.nex’-files and ‘.xml’-files for fossil calibrated analysis in BEAST [7]. Resulting topologies can be found in ‘.new’-file formats. A list of specimens and samples together with sampling time and locality is provided in Table 1 in [1]. A ‘.nex’-file [Dinematichthyidae_map_morph_05.nex] that holds 15 traits and morphological characters for the 30 species of Ophidiiformes is also included and can be opened in Mesquite v.3.04 [10].

## 2 Experimental design, materials and methods

### 2.1 Input files for inferring phylogenetic relationship in Bythitidae and Dinematichthyidae

Detailed descriptions of how DNA sequences were obtained and analyzed can be found in the main material and methods section by [1], associated with this article. The data used for inferring the relationship between Bythitidae and Dinematichthyidae [1] comprise one nexus-file, one BEAUti-file and two ‘.xml’-files with sequence alignments of mtDNA and nDNA markers inferred from 30 tissue samples, together with the sequences from the 23 outgroup representatives [mb_ophidiiform.nex] (see Tables 1 and 2 by [1]) and resulting topologies in ‘.new’-file format. The input-data matrix provided in [mb_ophidiiform.nex] was used in MrBayes [6], the settings (i.e. data partitioning and nucleotide substitution models used for this analysis) is implemented in this input-file [mb_ophidiiform.nex]. The phylogeny inferred from the MrBayes analysis is presented in Fig. 2 by [1] and can also be explored as a ‘.new’-file [mb_ophidiiform.nex.con.new] provided in this zipped supplementary material. The MrBayes inferred topology was made completely bifurcating in Mesquite v. 3.04 [10] and used as a starting tree in the ‘.xml’-files prepared in BEAUti v.1.8.0 [7]. Both the BEAUti-file [beast_ophidiiform_input.beautiv180] and the input-‘.xml’-files [beast_ophidiiform.xml] and [beast_ophidiiformE.xml] for BEAST are provided in the zipped supplementary data file. The taxon sets and fossil calibrations applied in the ‘.xml’-files are listed in Table 3 by [1] and can also be found in both the ‘.xml’-file [beast_ophidiiform_v04.xml] and in the BEAUti-file [beast_ophidiiform_input_04.beautiv180]. These input-files also hold information on the partitioning schemes and the substitution models applied in the fossil-calibrated analysis. The resulting time-calibrated topology is presented in Fig. 3 by [1] and can also be explored in the ‘.new-file’ [beast_ophidiiform_v04_02.tre.new] provided.

All supplied supplementary data files for MrBayes [6] and BEAST [7] can be analyzed directly in the appropriate software following the methods described in the material and methods section provided by [1].

### 2.2 Input files for reconstructing ancestral states in Bythitidae and Dinematichthyidae

The file with traits and morphological characters [Dinematichthyidae_map_morph_05.nex] can be opened in Mesquite 3.04 [10] and the used for mapping traits on the consensus tree derived from the BEAST analysis of the ‘.xml’-input file for BEAST v.1.8.0 [beast_ophidiiform_v04.xml]. The likelihood for each character and trait can be reconstructed by using all the 9000 trees obtained from the BEAST analysis after burnin, and tracing reconstructed states across all 9000 trees as described by [1].

## Footnotes

Appendix A Supplementary data associated with this article can be found in the online version at doi:10.1016/j.dib.2016.05.055.
